# Zanubrutinib for the treatment of lymphoid malignancies: Current status and future directions

**DOI:** 10.3389/fonc.2023.1130595

**Published:** 2023-03-23

**Authors:** Anna Wolska-Washer, Tadeusz Robak

**Affiliations:** ^1^ Department of Experimental Hematology, Medical University of Lodz, Lodz, Poland; ^2^ Department of Hematooncology, Copernicus Memorial Hospital, Lodz, Poland; ^3^ Department of Hematology, Medical University of Lodz, Lodz, Poland; ^4^ Department of General Hematology, Copernicus Memorial Hospital, Lodz, Poland

**Keywords:** zanubrutinib, chronic lymphocytic leukemia, DLBCL, mantle cell lymphoma, marginal zone lymphoma, Waldenstrom macroglobulinaemia

## Abstract

Zanubrutinib (BGB-3111, Brukinsa^®^, BeiGene) is a next-generation irreversible inhibitor of Bruton’s tyrosine kinase (BTK), developed by BeiGene in 2012 for the treatment of B-cell malignancies. It was designed to minimize off-target inhibition of TEC- and EGFR-family kinases. Zanubrutinib is more selective than ibrutinib for BTK versus EGFR, FGR, FRK, HER2, HER4, ITK, JAK3, LCK, BLK and TEC. In addition, compared to ibrutinib, zanubrutinib has improved oral absorption and better target occupancy. Zanubrutinib demonstrated a lower incidence of off-target toxicities and reduced severity than ibrutinib. Moreover, zanubrutinib is similar to acalabrutinib, with less activity against TEC and ITK. The preliminary phase 1 results suggest that zanubrutinib has clinical activity and the drug is well tolerated in patients with B-cell lymphoid malignancies. Recent clinical trials have found it to demonstrate excellent efficacy and good tolerability in patients with chronic lymphocytic leukemia (CLL), Waldenstrom macroglobulinemia (WM) and mantle cell lymphoma (MCL). In recent phase 3 studies, zanubrutinib was compared with ibrutinib in patients with relapsed/refractory (R/R) MW and RR CLL. In both trials, zanubrutinib was found to demonstrate clinically meaningful advantages in safety and tolerability over ibrutinib; in particular, it was associated with a lower risk of atrial fibrillation/flutter and major bleeding events. In the recent SEQUOIA study, comparing zanubrutinib with bendamustine and rituximab (BR) in patients with previously untreated CLL, zanubrutinib significantly improved progression-free survival versus BR, with an acceptable safety profile consistent with previous studies. Zanubrutinib also demonstrated good activity and tolerability in patients with R/R MCL, marginal zone lymphoma and follicular lymphoma. Trials examining the efficacy and safety of the combination of zanubrutinib with obinutuzumab venetoclax and other drugs are ongoing. This review summarizes the clinical efficacy and safety of zanubrutinib in lymphoid malignancies.

## 1 Introduction

Bruton’s tyrosine kinase (BTK) is a major source of pathogenic signaling in numerous lymphoproliferative malignancies (1–12) (**Table 1**). The BTK gene encodes a 659-amino acid, protein-tyrosine kinase with several domains (Pleckstrin homology, Tec homology, SRC homology, catalytic domain) (13). The name is attributed to Ogden C Bruton, who first reported that mutations of BTK and the resulting lack of appropriate signaling resulted in X-linked agammaglobulinemia with impaired B lymphocyte development and function (14). The activity of BTK has been found to be implicated in the development and propagation of various B-cell lymphoid malignancies (15, 16). Therefore, BTK function blockage has become an attractive therapeutic option in B-cell malignancies, as well as autoimmune diseases (14).

**Table 1:** Clinical trials of zanubrutinib in B-cell lymphoid malignancies.

Trial/Reference No	Disease/Phase	No of pts	Treatment	Patient characteristics	Follow up [months]	ORR (CR)	PFS months	OS months	Any-grade AEs (%, >15% pts)	Grade ≥ 3 AEs (%)
BGB-3111-AU-003 (5)	MCL/1/2	32	Zanu	R/R mostly intermediate- (40.6%) and high-risk (31.3%); 70.5 years	18.8	84% (25%)	21.1	NE	Infection 69%, bleeding 56%, diarrhea 44%, contusion 37%, constipation 31%, URTI 31%, fatigue 25%, dyspnea 25%, rash 22%, muscle spasms 16%, pneumonia 12%	Infection 19%, anemia 12%, neutropenia 9%, pneumonia 9%, myalgia 9%, bleeding 9%, major hemorrhage 9%, thrombocytopenia 6%
BGB-3111-206 (6)	MCL/2	86	Zanu	R/R mostly intermediate- (45%) and high-risk (38%); 61 years	35.3	84% (78%)	33	NR	Neutropenia 46%, URTI 38%, rash 36%, thrombocytopenia 33%	Neutropenia 19%, pneumonia 13%, thrombocytopenia 7%, anemia 6%.
SEQUOIA – arm A and B (7)	CLL/SLL/3	241 vs. 238	Zanu (A) vs. BR (B)	TN	26.2	95% (7%) vs. 85% (15%)	NR (24-mo PFS 85% vs. 69%)	NR (24-mo OS 94% vs. 95%)	In arm A: bleeding 44%, contusion 19%, constipation 19%, URTI 17%, neutropenia 16%. In arm B: neutropenia 57%, nausea 32%, pyrexia 27%, rash 20%, anemia 19%, IRR 18%, fatigue 16%.	In arm A: neutropenia 12%, hypertension 6%, cardiac AEs 5%, bleeding 4%, pneumonia 2%, URTI 1%. In arm B: neutropenia 51%, thrombocytopenia 7%, hypertension 5%, cardiac AEs 4%, pneumonia 4%, pyrexia 4%, URTI 1%.
SEQUOIA – arm C (8)	CLL/SLL/3	110	Zanu	TN del(17p)	30.5	90% (6%)	NR (24-mo PFS 89%)	NR (24-mo OS 94%)	Bleeding 51%, URTI 21%, contusion 20%, arthralgia 20%, neutropenia 18%, cardiac AEs 16%, constipation 15%, nausea 15%, back pain 15%.	Neutropenia 15%, pneumonia 6%, bleeding 5%, cardiac AEs 5%, headache 2%, thrombocytopenia 1%, pyrexia 15, back pain 1%.
SEQUOIA – arm D (9)	CLL/SLL/3	35	Zanu + vene	TN del(17p)	11.2	97% (13%)	NR	NE	Diarrhea 14%, neutropenia 14%, fatigue 11%, nausea 11%, petechiae 11%.	Neutropenia 11%, diarrhea 6%.
ALPINE (10)	CLL/SLL/3	324 vs. 324	Zanu (A) vs. ibrutinib (B)	R/R	29.6	86% vs. 76%	NR vs. 34.2 mo	NR	In arm A: infections 71%, neutropenia 29%, hypertension 23%. In arm B: infections 73%, neutropenia 24%, hypertension 23%.	In arm A: infections 26%, neutropenia (21%), hypertension (15%), pneumonia 13% incl. COVID-19, COVID-19 7%, AF/F 2%. In arm B: infections 28%, neutropenia 18%, hypertension 14%, pneumonia incl. COVID-19 12%, COVID-19 8%, AF/F 4%.
BGB-311-AU-003 (5)	FL/2	33	Zanu	R/R high- and intermediate FLIPI 60%	32.8	36% (18%)	12-mo PFS 38%	NR (3-year 76%)	Bleeding 54%, URTI 27%, URI 24%, contusion 24%, nausea 24%, cough 24%, fatigue 21%, neutropenia 18%, anemia 15%.	Infections 30%, neutropenia 18%, anemia 15%, hypertension 6%, bleeding 6%.
NCT02569476 (11)	FL/1b	36	Zanu + obinutuzumab	R/R 39% rituximab-refractory	20	72% (39%)	25	NE	URTI 39%, contusion 28%, fatigue 25%, cough 22%, nausea 19%, rash 19%, thrombocytopenia 19%, diarrhea 17%, neutropenia 17%, petechiae 17%, cough 17%.	Neutropenia 14%, thrombocytopenia 6%.
ROSEWOOD (12)	FL/2	145 vs. 72	Zanu + obi (A) vs. Obi (B)	R/R after ≥ 2 lines of therapy	12.5	68% (37%) vs. 46% (19%)	27.4 vs. 11.2	NE	In arm A: thrombocytopenia 34%, neutropenia 27%, diarrhea 16%, fatigue 14%, constipation 13%, cough 12%, pyrexia 11%, dyspnea 10%. In arm B:	In arm A: neutropenia 22%, thrombocytopenia 14%, major bleeding 1%, AF/F 1%. In arm B:

NE, not estimable; NR, not reached; URTI, upper respiratory tract infection; URI, urinary tract infection; AF/F, atrial fibrillation/flutter; Zanu, zanubrutinib; BR, bendamustine and rituximab; Obi, obinutuzumab.

The first-in-class BTK inhibitor approved for use by the US Food and Drug Administration (FDA) was ibrutinib (PCI-32765, Imbruvica, Janssen), which was initially registered in November 2013 for relapsed/refractory (R/R) mantle cell lymphoma (MCL) and in February 2014 for R/R chronic lymphocytic leukemia (CLL)/small lymphocytic lymphoma (SLL) (17). Soon the indications also included Waldestrom macroglobulinemia (WM), marginal zone lymphoma (MZL) and recently chronic graft-versus-host disease (GVHD). Ibrutinib binds covalently to a cysteine residue at the 481 amino acid position on BTK and blocks the subsequent downstream signal transduction (18). However, ibrutinib is not a selective BTK inhibitor, and its off-target action results in a number of side effects (19). Its capacity to bind kinases with Cys481 residues and other without such domains leads to several specific side effects, i.e. skin rash (EGFR, SRC kinases), bleeding (BTK/Tec kinases), infectious complications (ITK kinases), headache and atrial fibrillation (SRC kinases) (20–23). The second generation of BTK inhibitors, acalabrutinib and zanubrutinib, have been designed to reduce the off-target activity of ibrutinib and yet enhance efficacy (24).

Zanubrutinib (BGB-3111, Brukinsa^®^, BeiGene USA, Inc., San Mateo, CA, USA) is an orally-available, second-generation BTK inhibitor (25, 26). It binds covalently to the same Cys481 residue of BTK as ibrutinib, but shows increased specificity with decreased inhibition of other kinases i.e. EGFR, HER2, ITK, JAK3, TEC, BMX and BLK (27). The maximum serum concentration is achieved two hours after a single oral administration, with the area under the curve (AUC) depending on the dose (28). The half maximal inhibitory concentration (IC50) was only 0.3 nM and the median BTK occupancy in the nodal samples was 95% with 320mg QD and 100% with 160mg BID. The latter dose was chosen for further investigation. This review presents the current state of knowledge regarding the use of zanubrutinib in lymphoid malignancies including MCL, WM, CLL/SLL and other diseases.

## 2 Zanubrutinib – approved indications

### 2.1 Mantle cell lymphoma

Zanubrutinib was first granted approval in November 2019 by the FDA for the treatment of patients with R/R MCL. The decision was based on the results of a phase 1/2 trial BGB-3111-AU-003 (NCT02343120) and a phase 2 clinical trial BGB-3111-206 (NCT03206970) (**Table 1**) (5, 6). The phase 1/2 trial enrolled 32 patients with mostly intermediate- and high-risk MCL, who received a total daily dose of 320mg of zanubrutinib (6). Median follow-up was 18.8 months and the median time on treatment was 15.4 months. The primary reason for treatment discontinuation was disease progression (31.3%) and adverse events (AEs; 25%). The overall response rate (ORR) was 84%, with complete remission (CR) achieved in 25% of patients, and a median time to response (CR and partial response (PR) of 2.8 months. It was found that 160mg twice-daily administration was slightly more effective than 320mg once-daily dosing. The median progression-free survival (PFS) was 21.1 months and overall survival (OS) was 83% and 64.4% at 12 and 24 months, respectively. The phase 2 clinical trial included 86 patients who had received a median of two prior lines of treatment and were mostly high- and intermediate-risk (5). At a median follow-up of 18.4 months, the median number of 28-day cycles of treatment was 19.3 and nearly 40% of patients had discontinued therapy. The main reasons were progressive disease (46%) and AEs (15.4%). The ORR was 84% with 68.6% of subjects achieving CR, and the median time to response was 2.7 months. Overall, the treatment proved safe and well tolerated in both the abovementioned trials. The most common AEs of any grade were diarrhea (15.1 – 48.8%), upper respiratory tract infections (31.3 – 34.9%), contusion (4.7 – 37.5%) and constipation (31.3%). The grade ≥3 AEs of special interest included anemia (5.8 – 12.5%), neutropenia (9.4 – 19.8%), infections (18.8%), pneumonia (9.4 – 10.5%), myalgia (9.4%), thrombocytopenia (4.7 – 6.3%), bleeding (1.2 – 9.4%), major hemorrhage (1.2 – 9.4%), hypertension (3.1 – 3.5%), atrial fibrillation/flutter (AF/F; 3.1%), and tumor lysis syndrome (TLS; 6.3%). A longer follow-up (median 35.3 months) did not reveal an increase of the incidence of AEs, with 45.3% of patients continuing the treatment (29). The ORR was 83.7% and CR of 77.9%. Subgroup analysis identified better outcomes in patients < 65 years old, classic histology vs. blastoid histology, *TP53* wild type, and Ki67 ≤ 30%. Median duration of response (DOR) was not reached. Median progression free survival (PFS) was 33 months and median OS was not reached. During the extended follow-up, no cases of AF/F, grade ≥3 cardiac AEs, second primary malignancies or tumor lysis syndrome (TLS) were reported. A pooled analysis of long-term outcomes showed that second-line treatment yielded greater benefits in terms of OS in R/R MCL compared to later-line zanubrutinib treatment (30). Given the data, zanubrutinib shows remarkable activity in a heavily-pretreated or elderly population. Standard first-line treatment regimens (R-CHOP, BR) for elderly patients ineligible for autoSCT give median PFS from 13 to 35 months. The outcomes in the ≥ 2-line setting are worse. Targeted agents i.e. BTKis combined with bcl-2 inhibitor and monoclonal antibodies might lead to favorable outcome, but still around 2 years of PFS (31).

Zanubrutinib is currently being evaluated in clinical trials in treatment-naïve (TN) MCL. A phase 3 study comparing zanubrutinib and rituximab treatment followed by zanubrutinib monotherapy to rituximab and bendamustine in transplant-ineligible patients started in 2019 and is planned to enroll 500 participants randomized 1:1 to two treatment arms (NCT04002297) (32). The rationale behind that combination was based on the outcome of patients with R/R MCL who received rituximab with ibrutinib (33). At a median follow-up of 16.5 months, the ORR was 88% with 44% CRs and 44% PRs. Zanubrutinib with its better specificity towards BTK does not hamper the rituximab-induced antibody-dependent cell-mediated cytotoxicity as much as ibrutinib, so such combination might be even more effective (34). Perhaps the addition of zanubrutinib to the standard of care R-CHOP/R-DHAP in younger newly-diagnosed patients could spare the necessity to consolidate the treatment with autologous stem cell transplantation (ASCT). The assumption is based on a randomized Triangle trial presented at ASH Annual Meeting in 2022 in 870 patients treated with ibrutinib as the addition to standard chemotherapy with or without ASCT, and followed by ibrutinib maintenance (35). The results demonstrated high efficacy with good tolerability of ibrutinib during the induction phase and maintenance without the ASCT with a 3-year failure-free survival (FFS) of 86% vs. 72% after the standard treatment followed by the ASCT. The analysis whether ASCT can be safely omitted in patients receiving BTKi is still ongoing.

Patients with MCL and *TP53* mutations fare worse with standard chemoimmunotherapy with a median OS of 1.8 years (36). In a multicenter, investigator-initiated phase 2 BOVen trial zanubrutinib was evaluated in combination with obinutuzumab and venetoclax in previously untreated *TP53* mutant MCL (36). At the time of presentation, 12 patients have been analyzed of the 25 total planned accrual. The grade 3 treatment-related AEs included infusion-related reaction (17%), neutropenia (8%) and elevation of transaminases (8%). Tumor lysis syndrome (TLS) was not observed. At a median follow up of 4 months (range 0 - 11 months), one patient has progressed and eleven patients remained on study in continued response. The preliminary results were promising, and eight out of ten evaluable patients achieved PET CR at cycle 3. Longer follow-up data are pending.

A phase 2 trial of treatment-naïve (TN) MCL young and fit patients (BRIDGE; NCT04736914), which started in China in 2021 examined the addition of zanubrutinib to three cycles of R-CHOP (rituximab, cyclophosphamide, doxorubicin, vincristine, prednisone) alternating with three cycles of R-DHAOx (rituximab, cisplatin, cytarabine, and dexamethasone) followed by autologous stem cell transplantation (ASCT) and zanubrutinib maintenance for two years; it is planned to enroll 47 patients in the study.

Another approach involved the use of zanubrutinib plus rituximab (ZR) induction therapy until achieving CR or up to 12 cycles, followed by 4 cycles of R-DHAOx chemoimmunotherapy and zanubrutinib maintenance for one year in 17 TN MCL patients (37). Preliminary results indicate a CR rate of 88% after four cycles of ZR, with all patients having negative MRD in the bone marrow. The most common AEs were thrombocytopenia, rash, neutropenia, subcutaneous hemorrhage, and fatigue. The MRD status was found to be consistent with the imaging findings and might become a predictor of response in future trials. However, the data needs further analysis. The improved safety profile of zanubrutinib monotherapy is currently being tested in a phase 2 trial in patients with B-cell lymphoid malignancies previously treated and intolerant to ibrutinib or acalabrutinib (NCT04116437) (**Table 2**). The inability to use anticoagulants or gastric acid-reducing medication concurrently with acalabrutinib is one of the inclusion criteria.

**Table 2:** Ongoing clinical studies with zanubrutinib in lymphoid malignancies.

Study title	Phase	Disease	No. of patients	NCT number	Study start
A Study Comparing Obinutuzumab and BGB-3111 Versus Obinutuzumab Alone in Treating R/R Follicular Lymphoma	2	R/R FL	217	03332017	Nov 2017
A Study of Zanubrutinib Versus Lenalidomide in Participants With Relapsed/Refractory Marginal Zone Lymphoma	3	R/R MZL	372	05100862	March 2022
Treatment of CD79B Mutant Relapsed/Refractory Diffuse Large B-Cell Lymphoma With Bruton Tyrosine Kinase Inhibitor Zanubrutinib	2	R/R DLBCL	66	05068440	August 2021
Zanubrutinib, in Combination With Lenalidomide, With or Without Rituximab in Participants With Relapsed/Refractory Diffuse Large B-Cell Lymphoma	1	R/R DLBCL	67	04.436107	September 2020
Zanubrutinib Monotherapy in Relapsed/Refractory Central Nervous System Lymphoma	NA	R/R CNS	20	05117814	September 2021
Prospective, Single-arm, Phase II Clinical Study of Rituximab, Lenalidomide, and Zanubrutinib Combination Regimen Followed by Immunochemotherapy in the Treatment of Elderly Patients With Newly-diagnosed Diffuse Large B-cell Lymphoma	2	TN DLBCL	48	05290090	May 2022
Zanubrutinib-based Induction and Maintenance Therapy in Young and Fit Patients With Untreated Mantle Cell Lymphoma	2	TN MCL	47	04736914	January 2021
A Study to Evaluate Efficacy and Safety of Zanubrutinib With R-CHOP in Newly Diagnosed Non-GCB DLBCL Patients With Double (MYC and BCL2) Expression	2	TN DLBCL	41	05189197	January 2022
Zanubrutinib and CAR T-cell Therapy for the Treatment of Recurrent or Refractory Aggressive B-cell Non-Hodgkin’s Lymphoma or Transformed Indolent B-cell Lymphoma	2	NHL	24	05202782	March 2022
Study of BGB-10188 as Monotherapy, and in Combination With Zanubrutinib, and Tislelizumab	1/2	NHL	150	04282018	February 2020
Zanubrutinib in Combination With R-CHOP (ZaR-CHOP) for Newly Diagnosed Diffuse Large B-Cell Lymphoma	1	TN DLBCL, RT DLBCL	24	04850495	November 2021

FL, follicular lymphoma; MZL, marginal zone lymphoma; DLBCL, diffuse large B-cell lymphoma; CNS, central nervous system; TN, treatment naïve; Mantle Cell Lymphoma, NHL, non-Hodgkin lymphoma; RT, Richter transformation.

### 2.2 Waldenström’s macroglobulinemia

Waldeström’s macroglobulinemia is a rare type of non-Hodgkin lymphoma (NHL) with a median age of 70 years at diagnosis (38). The majority of cases will present with the bone marrow as the primary site of involvement (62%). The treatment is administered in patients with active disease defined as i.e. worsening of cytopenias, spleno- or hepatomegaly, lymphadenopathy and general symptoms, as WM is a type of indolent lymphoma (39). The cells of WM present a constitutive activation of the B-cell receptor (BCR) signaling with over 90% of patients harboring the activating mutations in the adaptor protein MYD88 (*MYD88^L265P^*), and another 30% to 40% of patients having an additional mutation in the chemokine receptor CXCR4 (*CXCR4^WHIM^)* (40). Patients with wild-type MYD88 and/or mutated CXCR4 have poorer outcomes. Other mutations downstream of BTK and interleukin-1 receptor-associated kinase (IRAK) induce nuclear factor kB (NF-kB) activation and resemble those found in diffuse large B-cell lymphoma (41).

Probably the greatest single-agent activity in WM is demonstrated by the Bruton’s tyrosine kinase inhibitor ibrutinib (42, 43). The final results of the iNNOVATE phase 3 clinical trial in rituximab-sensitive patients with TN WM or R/R WM showed that median PFS was not reached in the ibrutinib plus rituximab group, compared to 20.3 months for those on rituximab only (44). The results were independent of prior treatment status, *MYD88* and *CXCR4* mutation status, or key patient characteristics. A substudy analysis found that single agent ibrutinib administered to patients who failed to achieve any response or relapsing < 12 months after their last rituximab-based treatment demonstrated an overall median PFS of 39 months (45). Patients with MYD88^L265P^/CXCR4^WHIM^ and MYD88^L265P^/CXCR4^WT^ subtypes achieved median PFS of 18 months and not reached, respectively. Therefore, BTK inhibition is an interesting option in WM. The first-in-human phase 1/2 study of zanubrutinib was BGB-3111 AU-003 in patients with TN or R/R WM (46). The dose chosen was 160mg twice daily, based on the almost complete BTK occupancy. The study included 24 TN and 53 R/R BTKi-naïve WM patients. With respective median follow-ups of 23.5 and 36 months, the ORR was 100% in TN WM and 93.9% in R/R WM, and the major response rate (MRR; PR or better) was 87.5% and 79.6%, respectively. Median time to response was 2.8 months in both groups and median PFS was not reached in either group. However, there was no correlation between the depth of response with PFS. The treatment was well tolerated, and AEs included grade 1 contusion (32.5%), grade ≥3 neutropenia (15.6%), grade 3 anemia (9.1%), major hemorrhage (3.9%), AF/F (5.2%), grade 3 hypertension (3.9%), any-grade headache (18.2%), and grade 3 diarrhea (2.6%).

A direct comparison between ibrutinib and zanubrutinib was made in a phase 3 ASPEN trial in patients with R/R *MYD88^L265P^* WM or TN WM ineligible for standard immunochemotherapy (47). The primary end point was CR and a very good partial response (VGPR). A total of 201 patients were randomized. At a median follow-up of 19.4 months, a higher, but not statistically significant, rate of VGPR was observed for zanubrutinib vs ibrutinib (28% vs 19%, respectively), although no CRs were reported. Median time to achieve VGPR was 5.6 months for zanubrutinib vs 22.1 months for ibrutinib in TN WM, and 4.7 (zanubrutinib) vs 5.1 months (ibrutinib) for R/R MW. Median duration of response (DOR) and PFS were not reached, and approximately 85% of patients were progression free at 18 months. Zanubrutinib induced major responses in patients with *CXCR4^WHIM^* in 3.1 months vs 6.6 months for ibrutinib. Perhaps the most important result was the sustained reduction of the IgM concentration in favor of zanubrutinib which may lead to lower WM-related morbidity (48). A substudy analysis of the ASPEN trial was performed in a group of patients with wild-type MYD88 (*MYD88^WT^*) or inconclusive MYD88 (49). The study included 28 subjects in total: five TN and 23 R/R. At a median follow-up of 17.9 months, PFS and OS have not been reached. The ORR among the confirmed *MYD88^WT^* cases was 81%, with 27% desmonstrating VGPR and 50% of major responses. The most common any-grade AEs reported in the zanubrutinib arm were neutropenia in 29%, upper respiratory tract infection in 24%, and diarrhea in 21% of patients. The most common any-grade AEs in the ibrutinib arm were diarrhea (32%), upper respiratory tract infections (29%), contusion (24%), and muscle spasms (24%). Grade ≥3 hypertension and pneumonia were seen more often for ibrutinib vs. zanubrutinib. Grade ≥3 neutropenia was observed more often for zanubrutinib. Any grade and grade ≥3 AF/F was reported in 15% and 4% for ibrutinib compared to 2% and 0% for zanubrutinib. Zanubrutinib showed superiority in *MYD88^WT^CXCR4^WHIM^* cases over ibrutinib. Neither of the studied BTKis achieved CR which reflects the difficulty in eradicating WM even with targeted treatment. Perhaps combination therapies will help to obtain deeper responses. Venetoclax has been found to sensitize WM cells *in vitro* to BTKi by lowering the bcl-2-mediated signaling (50).

Zanubutinib was investigated in a combination with ixazomib and dexamethasone in a phase 2 clinical trial in twenty newly-diagnosed symptomatic patients with WM (NCT04463953) (51). The majority (70%) of patients were less than 65 years old, and had intermediate- (60%) and high-risk (20%) disease. The ORR was 100% and the median time to response was 3.7 months. All patients recovered their normal hemoglobin concentration, and measurable residual disease (MRD) was undetectable in two patients. The median follow-up was 12.3 months (range 5.4 – 23.4) and all subjects were alive at study data analysis. The treatment was well tolerated with few grade ≥ 3 AEs (rash and neutropenia). The study will publish updated results.

Given the rarity of WM and its indolent nature, it is difficult to demonstrate meaningful results as they are usually based on PFS. Standard therapies (BR, BDR, CDR) give PFS of about 6 years (52). Targeted therapies may offer MRD negativity so probably combination treatment options are the key to reach for long PFS. Other measurements such as the MRD status are therefore needed to predict the long term outcome.

### 2.3 Marginal zone lymphoma

Marginal zone lymphoma (MZL) represents about 5% to 15% of all non-Hodgkin lymphomas in the Western world (53). The survival of MZL cells depends on constitutive BCR signaling, and is mediated through a net of multiple kinases, including BTK (54). Ibrutinib proved its efficacy in MZL and has become a drug of choice in R/R MZL (55). In September 2021, zanubrutinib received accelerated approval for R/R MZL for patients who have received at least one anti-CD20-based treatment (56). The approval was based on two clinical trials. A phase 1/2 multicenter study BGB-3111-AU-003 (NCT02343120) included 20 patients with R/R MZL, with nine extranodal MZL, five nodal MZL, and six splenic MZL subtypes (57). The majority of patients had received rituximab-based monotherapy and the median number of lines of therapy was two. The most commonly reported AEs were diarrhea, contusion and rash (in 35%), upper respiratory tract infection and neutropenia (in 30%), nasopharyngitis and pyrexia (in 25.0%), and sinusitis, nausea, fatigue, and musculoskeletal pain (in 20%). The most common grade ≥ 3 AEs were neutropenia (20%), anemia (15%), thrombocytopenia (10%), and pyrexia (10%). Bleeding events of any grade were reported in 12 patients (60%). Grade ≥3 bleeding or CNS bleeding of any grade were reported in 2 patients. Grade 3 hypertension was reported in one patient, and atrial fibrillation/flutter was not observed. The ORR was 80% with CR 20%. The best ORR was achieved in nodal MZL (100%), compared to extranodal MZL (88.9%) and splenic MZL (50%). Median time to response was 2.8 months. At a median follow-up of 33.8 months, the estimated PFS and OS rates at 24 months were 72% and 83.9%, respectively. In a phase 2 MAGNOLIA trial (BGB-3111-214), a total of sixty-eight patients with R/R MZL were enrolled (58). With a median follow-up of 15.7 months, the ORR was 68.2% with CR 25.8%, and the ORR was similar among all MZL subtypes. The ORR in a group of elderly patients (≥75 years) was 94.4%. The median DOR and PFS were not reached, except in patients with increased LDH whose PFS was 15.5 months. Overall survival at 12 months was 95.3%. The most common AEs were diarrhea (22.1%), contusion (20.6%), constipation (14.7%). Any-grade neutropenia was reported in 13.2% of patients and grade ≥ 3 neutropenia was found in 10.3% of subjects. No patients experienced any major bleeding, and grade 3 atrial fibrillation and grade 2 atrial flutter were reported in two patients. In the final analysis of the trial, with a median follow-up of 28 months and a median duration of 24 months, 66 patients were evaluable for efficacy (59). The ORR was 64%, 76%, 66.7% and 50% in extranodal, nodal, splenic, and unknown subtypes, respectively. The CR rate ranged from 8.3% for splenic to 40% for extranodal MZL. The most common AEs were bruising (23.5%), diarrhea (22.1%), constipation (17.6%), arthralgia (14.7%), pyrexia (14.7%), upper respiratory tract infection (13.2%), abdominal pain and back pain (each 11.8%). The most common grade ≥3 AEs were neutropenia (8.8%) and COVID-19 pneumonia (5.9%). Patients who achieved good responses continue in a long-term extension study (NCT04170283). Despite the low rate of complete remissions in patients with MZL, zanubrutinib achieves high PFS and is a valuable option especially in this usually older population of patients. One must bear in mind possible side effects with long-term duration treatment and potential drug interactions.

### 2.4 Chronic lymphocytic leukemia/small lymphocytic lymphoma

Zanubrutinib proved successful as monotherapy in a series of phase 1/2 studies (BGB-3111-1002, BGB-3111-AU-003, BGB-3111-205) in TN and R/R CLL/SLL patients (28, 60, 61). A pooled analysis of the data of 211 patients (19 TN, and 192 R/R) identified an ORR of 95.4% (100% vs. 91% in the TN compared to the R/R group) (62). With a median follow-up of 12.9 months to 30.5 months, patients with one prior line of treatment achieved a 98.9% ORR, compared to 90.7% ORR for those with >1 prior therapies. Comparable efficacy was observed, irrespective of high-risk genomic aberrations, with a response being achieved by 86% to 100% of patients with del17p/TP53mut and 82% with unmutated IGHV. The median time to response was 2.8 months. The majority of responses were PRs (from 66.7% to over 80% of responses). The treatment was well tolerated and the most common all-grade AEs included neutropenia (up to 69.2%), thrombocytopenia (up to 42%), anemia (up to 30%), petechiae, purpura, or contusion (up to 35% in total), hematuria (up to 40%), and upper respiratory tract infection (up to 45%). The most common treatment-emergent grade ≥ 3 AEs were neutropenia (up to 44%), thrombocytopenia (up to 15.4%), and upper respiratory tract infection (up to 9.9%). Atrial fibrillation/flutter was reported in four patients overall in the three analyzed trials (62).

Given the promising results of the initial early phase trials, zanubrutinib was further studied in two phase 3 clinical trials in TN (SEQUOIA) and R/R (ALPINE) CLL patients (**Table 1**) (7, 8). The outcomes of these trials led to the approval of zanubrutinib by the FDA in January 2023 for the treatment of adult TN and R/R CLL/SLL patients. However, neither of them studied zanubrutinib in younger, fitter patients. Zanubrutinib is already present among the recommended therapeutic options regardless of the risk factors by the German Society for Hematology and Medical Oncology (DGHO; https://www.dgho.de/). The SEQUOIA study included 590 patients unsuitable for fludarabine-cyclophosphamide-rituximab treatment because of age ≥ 65 years, comorbidities assessed as more than six points on the Cumulative Illness Rating Scale (CIRS), creatinine clearance less than 70mL/min, or medical history of frequent infections (7). Between October 2017 and July 2019, patients without del(17p) were randomized to receive either zanubrutinib monotherapy or rituximab with bendamustine (BR), and subjects with del(17p) were assigned to a third arm with zanubrutinib monotherapy. The estimated 24-month PFS was 85.5% with zanubrutinib and 69.5% with BR, and no difference in OS between the two groups. No significant difference in PFS was observed in patients with mutated IGHV, and zanubrutinib was as effective as BR among patients with SLL or mutated *TP53*. The ORR was 94.6% with zanubrutinib, and 85.3% with BR, and most responses were PRs. Richter transformation occurred in 2% and less than 1% of patients in the zanubrutinib and the BR group. Seven and six percent of patients had died at data cutoff. In patients with del(17p), 14% had progressed or died at the median follow-up of 30.5 months, but the median PFS was not reached. The estimated 24-month PFS was 88.9%, and the estimated 24-month OS was 93.6%. The most common grade ≥ 3 AEs in both groups were neutropenia (11% to 15% with zanubrutinib and 58% with BR) and infections (16% with zanubrutinib and 19% with BR). Adverse events of special interest with zanubrutinib were also reported: AF/F in 3%, major bleeding events 5% to 7% (compared to 2% with BR) and second primary malignancies of any grade in approximately 13% to 22% (compared to 9% with BR). The concomitant use of anticoagulants was not prohibited. The zanubrutinib group, excluding patients with del(17p), experienced 16 deaths during the study, compared to 14 with the BR group. No sudden deaths were reported. An analysis of patient-reported outcomes at weeks 12 and 24 of therapy found that zanubrutinib treatment significantly improved general health score, physical and role functioning, and reduced diarrhea, fatigue and nausea/vomiting, compared to BR (63). However, one has to bear in mind that the patients randomized to zanubrutinib continued the treatment while BR patients would have completed the therapy at week 24.

Treatment with BTK inhibitors in continuous monotherapy may not be optimal, as most responses are PRs and only the minority of patients achieve CR (64). There are long-term safety concerns including cardiac events and the risk of rapid progression following BTKi cessation (65, 66). Some patients might find the ongoing need for therapy unacceptable and difficult to comply in real-life scenarios. One possible solution is to combine BTKis with other targeted therapeutic options to induce deeper responses that will allow for treatment discontinuation once appropriate level of disease control has been achieved, currently indicated by minimal residual disease (MRD). Given the excellent outcome of patients with del(17p) on zanubrutinib monotherapy, a non-randomized arm D of the SEQUOIA study was added for TN CLL patients and del(17p) (9). The treatment involved an initial three months of zanubrutinib followed by combination treatment with venetoclax up to 12-24 months, depending on the outcome including MRD status. At the first data cutoff in June 2021, 35 patients were enrolled and 31 reached the initial efficacy assessment. The ORR was 96.8%, with one patient having PD on combination therapy. The regimen was well tolerated with mostly neutropenia and diarrhea, and one worsening of preexisting AF. The trial was yet too immature to give data on the MRD.

In the second phase 3 ALPINE non-inferiority trial comparing zanubrutinib to ibrutinib in patients with R/R CLL/SLL, 652 patients were enrolled between November 2018 and December 2020 (10). The published interim analysis included 415 patients randomized to receive zanubrutinib (n=207) or ibrutinib (n=208). At a median follow-up of 15 months, a significantly higher ORR was observed with zanubrutinib compared to ibrutinib (78.3% vs. 62.5%, respectively). In patients with unfavorable risk factors: the ORR was 80.5% for zanubrutinib vs. 50% in del(17p)/TP53mut, 83.6% vs. 69.1% in del(11q). The PFS at 12 months was 94.9% with zanubrutinib and 84% with ibrutinib, also favorable for zanubrutinib in a subgroup with del(17p)/*TP53*mut (91.5% vs. 74.4%). Treatment discontinuation due to AEs was observed in 7.8% of patients on zanubrutinib compared to 13% on ibrutinib, and infections were the most common reason (2.9% in each arm). Adverse events of special interest reported in zanubrutinib and ibrutinib groups were: AF/F (2.5% vs. 10.1%), anemia (13.2% vs. 15.5%), major hemorrhage (2.9% vs. 3.9%), hypertension (16.7% vs. 16.4%), secondary primary malignancies (8.3% vs. 6.3%). With a median follow-up of 29.6 months at the final analysis per protocol, the PFS rate at 24 months was 78.4% for zanubrutinib and 65.9% for ibrutinib (10). Median PFS with zanubrutinib was not reached, and median PFS with ibrutinib was 34.2 months. Zanubrutinib also showed benefit in high-risk patients with longer PFS (HR: 0.52 [95% CI:0.30-0.88]). Moreover, patients who received zanubrutinib monotherapy reported improved key health-related quality of life endpoints including general health, physical functioning, fatigue, and diarrhea (67).

A phase 1 trial with zanubrutinib combined with obinutuzumab, administered until unacceptable toxicity or disease progression, was conducted in a cohort of 45 TN or R/R CLL/SLL patients (11). Among patients with CLL/SLL there were 49% with unmutated IGHV, 41% with del17p)/TP53mut, 26% with del(11q) and 23% with a complex karyotype. With a median follow-up of 29 months, the ORR was 100% for TN CLL/SLL, and 92% for R/R CLL/SLL patients, including 30% and 28% of CRs, respectively. In high-risk patients the ORR was 100% (TN) and 80% (R/R), with 50% (TN) and 20% (R/R)reaching CR. The median PFS was not reached, and the estimated event-free rate at 24 months was 90.4%. Three out of six TN patients who achieved CR were also MRD negative at the 10^-4^ detection limit. The most common grade ≥ 3 AEs were neutropenia (31.1%), pneumonia (8.9%), thrombocytopenia (6.7%). There were no cases of AF/F or major hemorrhage. Neutropenia occurred more often during the combination of ibrutinib and obinutuzumab in the iLLUMINATE study (36% of grade ≥ 3) (68). The question remains whether the combination of zanubrutinib and obinutuzumab will improve PFS, as no such advantage has been found for combined ibrutinib and rituximab therapy over ibrutinib monotherapy (69). Zanubrutinib is not yet approved for the treatment of CLL/SLL. However, due to its potency and selectivity it has already become one of the recommended options by the NCCN Guidelines Version 1.2023. Chronic Lymphocytic Leukemia/Small Lymphocytic Lymphoma.

A different approach is currently being studied in a phase 2 clinical trial of the combination treatment with zanubrutinib, obinutuzumab and venetoclax, administered according to the MRD status in TN CLL/SLL patients (70). Thirty-nine patients were enrolled in this study. After a median follow-up of 25.8 months, 33 of 37 (89%) evaluable patients had undetectable MRD in the peripheral blood and bone marrow below the 10^-4^ limit, meaning that therapy could be stopped, after a median of ten cycles. After a median 15.8 months of observation after stopping treatment, 31 out of those 33 patients were still MRD-negative. With a median duration of treatment of 10 months, this combination was well tolerated with the most common all-grade AEs: thrombocytopenia (59%), fatigue (54%), neutropenia (51%; grade ≥ 3 18%), bruising (51%). The trial is currently recruiting patients with TP53-mutated MCL (NCT03824483) (**Table 2**).

The MRD assessment and/or the idea of guiding the therapy duration according to the MRD status in CLL is currently being studied in multiple trials. Resistance to targeted drugs may become an issue with subsequent relapses. Intermittent administration of therapy when treatment is stopped once the response has been achieved might prevent the otherwise inevitable emergence of drug resistance (see https://www.isrctn.com/ISRCTN51675454).

## 3 Zanubrutinib in other lymphoid malignancies

In addition to MCL, VM and CLL/SLL zanubrutinib has a potential in the treatment of other lymphoid malignancies including diffuse large B-cell lymphoma (DLBCL) and follicular lymphoma (FL).

### 3.1 Diffuse large B-cell lymphoma

Diffuse large B-cell lymphoma is one of the most common lymphoma subtypes in the adult population and its incidence increases with age (71). In addition, increasing age is associated with a higher incidence of comorbidities and inability to treat patients with sufficient intensity i.e. R-miniCHOP (72, 73). Bruton’s tyrosine kinase was found to be expressed in a substantial number of cases, especially in the non-germinal center B-cell-like (GCB) subtype (74). Moreover, BTK expression correlated positively with higher international prognostic index (IPI), and worse outcome after treatment with rituximab plus cyclophosphamide, doxorubicin, vincristine and prednisone (R-CHOP). A chemotherapy-free regimen was studied in a phase 2 trial with the BTKi ibrutinib, combined with lenalidomide and rituximab (iR2) (75). In a group of 30 treatment-naïve unfit or frail patients over 75 years of age, the ORR was 66.7% with 56.7% of CRs. However, there was a 10% incidence of atrial fibrillation with the majority of cases being grade 3. Zanubrutinib was studied as monotherapy in a phase 2 study in 41 patients with R/R non-GCB DLBCL after a median of two prior treatment lines (76). At a median follow-up of 6.8 months, the ORR was 29.3% with 17.1% CR. Median PFS and OS were 2.8 and 8.4 months, respectively. The most common treatment emergent grade ≥ 3 AEs were neutropenia (n=3), pneumonia (n=2), abdominal (n=2), and death of unknown cause (n=2). There were no cases of AF/F, major hemorrhage or tumor lysis syndrome. A SMART study examined the effect of zanubrutinib combined with lenalidomide and rituximab as the sole 21-day regimen for newly-diagnosed elderly patients (6 – 8 cycles), or as the initial 2 - 3 cycles followed by chemotherapy, if the patient was unable to tolerate chemotherapy at the beginning, as the so called SMART-START approach (77). Median age in the SMART arm was 82, compared with 67 in the SMART-START arm. Eleven out of 31 patients were on zanubrutinib, 16 used ibrutinib, and four were on olerabrutinib. Chemotherapy regimens included: R-CHOP, R-DAEPOCH, R-Gmox/RminiCHOP. In the whole study group, the SMART and the SMART-START groups demonstrated respective ORR values of 87.5% and 92.3%, and respective OS of 89% and 91% at a median follow-up of 15.4 months. The median number of SMART cycles was 5 with a median time to response of 1.2 months. In the SMART-START arm, 23% of patients achieved CR before chemotherapy. The study was retrospective and therefore might not provide objective data. A prospective trial with a ‘gentle initiation’ phase was studied in a group of 26 treatment-naïve elderly patients with DLBCL and a median age of 76 years (78). Patients received two cycles of zanubrutinib, lenalidomide and rituximab (ZR2), followed by four cycles of RCHOP. The outcome of the initial phase was 100% ORR (35% CR and 65% PR) regardless of the lymphoma subtype (non-GCB vs. GCB DLBCL). The treatment was well tolerated and proved remarkably successful in an elderly population with DLBCL. The most common all-grade AEs were neutropenia and anemia (both 38.5%), and thrombocytopenia (nearly 35%). At a median follow-up of 9.8 months, the median PFS and OS were not reached. A few patients continued ZR2 without the additional immunochemotherapy and achieved remissions at the end of the planned treatment schedule. The latter finding opens a new era for unfit patients ineligible for intensive chemotherapy who have limited options of therapy. Further studies are warranted, given the promising results of zanubrutinib in this patient population.

A phase 3 PHOENIX study evaluated the efficacy of ibrutinib combined with R-CHOP in the TN non-germinal center DLBCL (79). In a subpopulation of patients < 60 years of age, ibrutinib plus R-CHOP improved PFS and OS (HR 0.56 and 0.33, respectively); however, no such effect was demonstrated in older patients, with the combination treatment leading to higher toxicity. The ORR was similar in both treatment arms, with a slightly higher CR rate with ibrutinib (71.2% vs. 69.9%). It is necessary to mention that the analysis in the younger subpopulation was performed *post hoc*. Zanubrutinib plus R-CHOP showed improved outcomes in forty-four patients with DE DLBCL in a single-arm phase 2 study (80). The response rate after six cycles of ZR-CHOP was 68% CRs, 2.3% PRs and 6.8% progressive disease (PD). Ten patients died during the study and five patients progressed during or soon after the completion of the treatment. The toxicities were manageable, and grade ≥ 3 AEs were similar or less frequent than in the PHOENIX study. Promising results were also demonstrated in a cohort of R/R DLBCL patients with zanubrutinib in combination with lenalidomide in a phase 1 study (81). Another approach was studied in a multicenter phase 2 trial of zanubrutinib and lenalidomide added to R-miniCHOP in a population of elderly patients with non-GCB DLBCL (82). Sixteen patients were eligible for evaluation. The ORR was nearly 94% with 62.5% CRs. The most common AEs were neutropenia and thrombocytopenia. Zanubrutinib might become a potent addition to either reduced-intensity chemotherapy as in mini-CHOP in TN DLBCL or R/R DLBCL with otherwise dismal prognosis.

Three formulations of the chimeric antigen receptor T cell (CAR-T) therapy, axicabtagene ciloleucel, tisagenlecleucel, and lisocabtagene maraleucel, have been approved by the FDA for the treatment of relapsed/refractory DLBCL patients (83). The ZUMA-7, BELINDA, and TRANSFORM phase 3 clinical trials showed significant efficacy of CAR-Ts in the second line of treatment, and challenged the second line standard of care (SOC) with autologous stem cell transplantation. Median PFS significantly improved after CAR-Ts in ZUMA-7 and TANSFORM trials compared to SOC (14.7 and 14.8 months vs. 3.7 and 5.7 months, respectively). BELINDA trial failed to demonstrate superiority of CAR-Ts over SOC, which has been explained by a longer waiting period before the actual CAR-T infusion etc. There is still place for SOC with autoSCT procedure, however it might not be plausible for unfit or frail patients. Moreover, CAR-T therapy carries risk of complications such as prolonged cytopenia, cytokine release syndrome, relapse, and the timely availability or the quality of patient’s lymphocytes might become a hurdle (84). The place of BTK inhibitors in this setting is yet to be determined, and the question whether they should come before or after CAR-Ts is open. Perhaps these agents, including zanubrutinib, could be administered concurrently as immune modifiers after CAR-T infusion, as well as synergizing with the anti-lymphoma effect. There is a single case report on zanubrutinib combined with an oral histone deacetylase inhibitor (HDACi), chidamide, in a 70-year old patient with DLBCL who relapsed 12 months after CAR-T therapy (85). He failed salvage chemotherapy with gemcitabine, dexamethasone and cisplatin, and suffered from progressive and symptomatic lung infiltration. The treatment with zanubrutinib and chidamide brought immense relief within two weeks and a CT scan confirmed CR two months into the treatment which was sustained at a 10-month follow-up. The CAR-T clone was not detected at the time of relapse and CD19 was still present on tumor cells; therefore, it was proposed that the effect was achieved due to the synergistic effect of zanubrutinib and chidamide, rather than augmentation of CAR-Ts. The exact mechanisms are yet to be determined. The tumor DNA analysis in the studied case detected the mutations in TP53 (47.6%) and NOTCH1 (35%), without mutations in MYD88, CD79b, EP300 or CREBBP. Chidamide was chosen based on its inhibitory effect on the BTK pathway signaling, decreasing mutant TP53 expression, and ease of administration (oral). This study showed potential in the tailoring of the combined targeted treatment based on the results of the mutational status in the signaling pathways in the tumor cells. Previous studies showed that *CD79B* mutation carries a higher risk for unfavorable outcome in patients with DLBCL (86). The preliminary results of a phase 2 study in 137 patients with *CD79A*/*CD79B*-mutant TN and R/R DLBCL found the addition of zanubrutinib to augment R-CHOP or salvage therapy (87). With a median follow-up of 6.4 months (range 2.6 – 24.5 months), 12 TN and 9 R/R patients were evaluable for response. There were 10 CRs and 2 PRs in TN DLBCL, and 5 CRs and 2 PRs in R/R DLBCL. The most common grade ≥3 AEs in TN and R/R cohort were: neutropenia (26.7% and 60%, respectively), anemia (26.7% and 60%, respectively), thrombocytopenia (26.7% and 50%, respectively) and infection (20% in both). This study is still ongoing and awaiting survival data in the future.

### 3.2 Follicular lymphoma

Follicular lymphoma is one of the most common non-Hodgkin lymphoma subtypes in the Western world (88). It affects mainly elderly patients and presents an indolent course with remissions and subsequent relapses (71). The current approach depends on the stage of the disease, but is based on anti-CD20 antibodies and classical chemotherapy like CHOP or bendamustine. Newer agents include targeting molecules such as BTK inhibitors, phosphatidylinositol-3-kinase (PI3K) inhibitors or lenalidomide (an immunomodulatory agent) (89). However, due to the indolent course of the disease and long follow-up, it is difficult to demonstrate an absolute benefit in terms of OS or even PFS. Therefore, a good approach might be either to maximally reduce the disease burden at the expense of the likelihood of AEs, or keep the disease under control with fewer side effects. Chemotherapy-free regimens might meet both the criteria when given in combination or monotherapy.

Zanubrutinib was administered as monotherapy in 33 patients with R/R FL in the BGB-311-AU-003 phase 2 trial (57). A median age was 63 years and most patients presented with intermediate/high FLIPI score (60.6%) and extranodal disease (54.5%). The median number of prior therapies was three. At a median follow-up of 33.9 months, median PFS was 10.4 months and median OS was not reached. The ORR was 36.4%, with 18.2% CRs and 18.2% PRs. Progressive disease was reported in 15.2% of patients. The median time to response was 2.7 months. Grade ≥ 3 AEs included neutropenia (18.2%), urinary tract infection (18.1%), anemia (15.2%), pneumonia (9.1%), hypertension (6.1%), abdominal pain (6.1%). No incidents of AF/F were observed. One patient experienced serious hematuria. Twelve patients (36.4%) had dose interruptions due to AE, and three patients (9.1%) discontinued zanubrutinib because of AE, with a median relative dose intensity of 97.9%. Better results were observed with a combination of zanubrutinib and obinutuzumab in a phase 1b trial in a total of 36 patients with R/R FL (11). A median age was 59 years of age and a median number of prior lines of therapy was 2. Thirty-nine percent of patients were refractory to rituximab. The median duration of zanubrutinib treatment was 20 months. The most common AEs were upper respiratory tract infection (39%), contusion (28%), fatigue (25%), cough (22%), diarrhea (17%), hypertension (8%), and major hemorrhage (3%). No AF/F was observed. The most common grade ≥ 3 AEs were neutropenia (14%) and thrombocytopenia (5.6%). The ORR at a median follow-up of 20 months was 72% with 39% CRs. Median PFS was 25 months. Half of patients discontinued the study because of progression, patient’s or investigator’s decision, and AE. The results of this study were favorable given the heavily-pretreated population.

The ROSEWOOD phase 2 randomized trial compared the efficacy and safety of zanubrutinib plus obinutuzumab (ZO) vs. obinutuzumab (O) alone in R/R FL patients (12). Two hundred and seventeen patients after ≥ two lines of therapy (including an anti-CD20 antibody and an alkylating agent) were randomized 2:1 to receive either ZO (n=145) or O (n=72). At a median follow-up of 12.5 months, the ORR was 68.3% with ZO and 45.8% with O, and the CR rate was 37.2% (ZO) vs. 19.4% (O). Median PFS was 27.4 months with ZO compared to 11.2 months with O. The ORR for 29 patients who crossed over to the ZO arm was 24.1%. The most common AEs in the ZO arm were thrombocytopenia (34.3%), neutropenia (27.3%), diarrhea (16.1%), fatigue (14.0%), constipation (13.3%), cough (11.9%), pyrexia (11.2%), and dyspnea (10.5%). Grade ≥3 AEs with incidence > 5% with ZO were neutropenia (22.4%) and thrombocytopenia (14.0%); incidence of atrial fibrillation was 0.7% and major bleeding was 1.4%. Zanubrutinib with obinutuzumab demonstrated superior efficacy to obinutuzumab alone in this heavily-pretreated population. Idelalisib proved its efficacy in a population of R/R FL patients who failed on their previous chemoimmunotherapy or relapsed within six months (90). Moreover, dual inhibition of PI3K and BTK signaling might overcome emerging resistant to these single agents. In a phase 2 study, a PI3K inhibitor, zandelisib, was administered with zanubrutinib in a group of 31 patients after a median of two previous lines of therapy (91). The ORR was 80% with 20% CRs, and the preliminary median PFS was 22.4 months with a median follow-up of 11.1 months. The depth of response was observed to increase over time. The spectrum of AEs was not different from those observed for single agents and did not lead to increased treatment discontinuation. However, idelalisib and other PI3K inhibitors might lead to serious complications including diarrhea due to colitis, liver enzyme elevation, pneumonitis, infections, and intestinal perforation in a significant number of patients (92). Idelalisib is no longer indicated for FL or SLL by the FDA (92). The international development of zandelisib has also been terminated and will only continue in Japan (93). The only PI3K inhibitor approved for FL by the FDA is copanlisib, but there are currently no ongoing trials of copanlisib in combination with zanubrutinib.

## 4 Conclusions and future directions

Zanubrutinib is a next-generation irreversible inhibitor of BTK developed by BeiGene in 2012 for the treatment of B-cell malignancies with a great activity and a higher specificity towards BTK. It has proved its efficacy in several non-Hodgkin lymphomas in monotherapy, as well as in combination with other targeted, chemotherapy-free agents. In our opinion, the future of zanubrutinib lies among combination therapies, either as a lead-in before the classical cytotoxic agents, or as an added modality with bcl-2 inhibitor, monoclonal antibodies, bi-clonal antibodies, or immunotoxins. Moreover, zanubrutinib might enhance the efficacy of CAR-T cell therapy in lymphoma. Aiming at deepening the response in indolent lymphomas with multi-agent regimens is currently being evaluated. Such approach enables MRD-guided limited duration of treatment and presumably prevents the development of resistance. On the other hand, such efficacious regimens carry the risk of adverse events that might only be tolerated and managed in fitter patients; even so, in such cases, the anticipated benefit would outweigh the inevitable risks.

## Author contributions

All authors listed have made a substantial, direct, and intellectual contribution to the work and approved it for publication.
